# Our Journal

**Published:** 1852-01

**Authors:** 


### THE WESTERN JOURNAL.

THIRD SERIES.—VOL. IX.—NO. I.

LOUISVILLE, JANUARY 1, 1852.

### OUR JOURNAL.

With this number commences the twenty-fifth volume of our Journal, which, if to the term of its existence here be added the twelve years it attained under the same name in another city, and under the direction of one who was for a long time its senior editor in this, is one of the oldest medical journals in our country. Twenty-four years is no mean age for such a work to reach in our Republic.

At the beginning of a new year and a new volume, it is fitting that we should return our thanks to our friends for the support which many of them have given to our enterprise from its inception, and in doing so we cordially express our best wishes that the year may be one of success and happiness to them all, and that they may live to see the in-coming of many a new year.

In looking back over our editorial lives, while we are conscious of having performed an amount of drudgery not easily appreciated by those who have had no experience in such matters, and of having aimed steadily at advancing the true interests of our profession, we are far from feeling a spirit of pride, or any disposition to take credit to ourselves for what we have accomplished. On the contrary we are free to admit, that we have never been satisfied with our performance, and that our

Journal has constantly fallen far short of what, in our estimation, a medical journal in Louisville should be. Neither in matter, style, nor dress; neither as to scholarship nor science, as to literary excellence or mechanical execution, has it been worthy of the profession in our day or of the arts even in our own backwoods. Long ere now we had hoped to have effected a decided improvement in, at least, the smallest of these particulars; but another year has come and finds us as far as ever from the high standard to which we have been aspiring. But we do not despair. We are resolved to persevere in our efforts, and hope at no distant day to see our work issued in a style that shall do credit to the profession, and be worthy of more than the steady encouragement it has received from so many patrons.

It is a fact fact not to be disputed, that the medical profession in our country is advancing in scientific attainment—that the body of medical men in the United States, growing to be one of the most numerous in any country, is increasingly a reading, inquiring body. The medical journals should reflect the progress of the profession. Their readers have a right to expect in them, not only more accurate science and improved modes of practice, but riper scholarship and more literary taste. For ourselves, we can only promise our best endeavors. We have to rely upon our brethren engaged in practice for contributions of a practical character. If such of them as are our readers will generally give us their experience in what is passing under their eyes, we promise that our journal shall be more interesting and useful than it has ever yet been.—We must have contributors if our work is to improve. We hereby call upon our readers, one and all, to do something with their pens this year for their profession. Our numbers must appear at the appointed season whether contributions come or not. If matter is not furnished, the editors must produce it themselves; and oft-times their case is like that of the poor play-actor who, in the midst of the snow-storm he was representing, found his stock of white paper exhausted and was compelled to eke out his mimic tempest with brown.

Circumstances beyond our control have delayed the appearance of our present number. We hope we shall not have to apologise soon again for such an occurrence. If we can promise nothing better, we hope, at least, to be punctual hereafter.

